# Utilization of Copper Flotation Tailings in Geopolymer Materials Based on Zeolite and Fly Ash

**DOI:** 10.3390/ma17246115

**Published:** 2024-12-14

**Authors:** Marija Štulović, Dragana Radovanović, Jelena Dikić, Nataša Gajić, Jovana Djokić, Željko Kamberović, Sanja Jevtić

**Affiliations:** 1 Innovation Center of the Faculty of Technology and Metallurgy in Belgrade Ltd., University of Belgrade, Karnegijeva 4, 11120 Belgrade, Serbia; mstulovic@tmf.bg.ac.rs (M.Š.); jdikic@tmf.bg.ac.rs (J.D.); ngajic@tmf.bg.ac.rs (N.G.); 2 Innovative Centre of the Faculty of Chemistry, University of Belgrade, Studentski Trg 12-16, 11000 Belgrade, Serbia; djokic@chem.bg.ac.rs; 3 Faculty of Technology and Metallurgy, University of Belgrade, Karnegijeva 4, 11120 Belgrade, Serbia; zeljko.kamberovic@tmf.bg.ac.rs (Ž.K.); sanja@tmf.bg.ac.rs (S.J.)

**Keywords:** mining waste, geopolymerization, molarratios, reuse, geochemical modeling

## Abstract

Copper flotation tailings (FTs), resulting from the separation and beneficiation processes of ores, are a significant source of environmental pollution (acid mine drainage, toxic elements leaching, and dust generation). The most common disposal method for this industrial waste is dumping. However, due to their favorable physical and chemical properties—the high content of aluminosilicate minerals (60–90%)—flotation tailings can be effectively treated and reused through geopolymerization technology, thereby adding value to this waste. The objective of this study was to evaluate the potential of utilizing the geopolymerization of FTs to produce sustainable materials. Geopolymers based on natural zeolite (NZ), sodium-modified natural zeolite (NaZ), and fly ash (FA) were prepared using 20%, 35%, and 50% of FTs, activated with a 10 M NaOH solution. The study investigated the influence of Ca/Si, Si/Al, and Na/Al molar ratios on the structural, thermal, and mechanical properties (XRD, TG/DTG and unconfined compressive strength, UCS), and contaminant immobilization (TCLP method) of geopolymers. Geochemical modeling via the PHREEQC program was employed to interpret the results. The findings indicated that the UCS value decreased in zeolite-based geopolymers as the content of FT increased due to the inertness of the tailings and the low calcium content in the system (Ca/Si ≤ 0.3), in contrast to the FA-based geopolymer. The highest UCS of 44.3 MPa was recorded in an FA-based geopolymer containing 50% flotation tailings, with optimal molar ratios of 0.4 for Ca/Si, 3.0 for Si/Al, and 1.1 for Na/Al. In conclusion, the geopolymerization process has been determined to be a suitable technological approach for the sustainable treatment and reuse of FTs.

## 1. Introduction

The increased production and application of non-ferrous metals (aluminum, copper, zinc, and lead) [1] in metallurgy, mineral processing, and other industries is accompanied by the production of significant amounts of solid waste such as slag [2], sludge [3], ash [4,5], and tailings [6,7].

The mining industry is generating an increasing quantity of tailings due to the greater demand for metals and minerals, as well as the adoption of improved mining and processing processes to exploit low-grade minerals and extract more value from them. Some reports indicate that around 19–25 billion tons of solid mining waste are generated annually worldwide, with 5–8 billion tons of tailings [8,9].

Flotation tailings are fine-grained residues from ore separation and beneficiation, i.e., waste that is created by extracting valuable products from ore. It has an inhomogeneous and complex composition and contains a mixture of minerals with relatively low quantities of valuable components and effluents with hazardous and toxic compounds created during size reduction and mineral processing [10,11,12]. These waste materials are typically disposed of in tailing dams in the form of slurry.

The improper disposal of mining waste can have severe negative environmental impacts. The presence of air and moisture leads to acid mine drainage (AMD) formation, the leaching of toxic elements, the pollution of surface water, soil contamination, dust generation, and the mobilization and occupying of cultivated land by mining tailings. The flotation method produces tailings with smaller particles and increases the risk of contaminant release [13,14].

It is possible to decrease the challenges associated with flotation tailings by considering the physicochemical properties of tailings, as well as the methods used for their treatment and storage. Moreover, to address the issue of environmental pollution caused by tailings, it is important to implement sustainable and green treatment methods and explore their potential applications in industry to minimize their quantity and add value to this type of waste.

Mining tailings should be viewed as a source of minerals to manage natural resources rationally. Many forms of tailings have the potential for recycling and reuse, including Mn-rich tailings, which can be utilized to manufacture building materials, ceramics, glass, and coatings; clay-rich tailings, which can be used in bricks, sanitary wares, porcelain, and cement production; iron ore tailings mixed with supplementary materials, such as fly ash and sewage sludge and used in construction industry; and bauxite-comprising tailings, which can be utilized as Al resources [15]. Moreover, a high content of aluminosilicate minerals in flotation tailings (from 60 to 90%), as a source of silicon, aluminum, and calcium ions, [10,15] is a good prerequisite for the reuse of this type of mining waste in the production of geopolymer materials.

Geopolymers are materials with specific mechanical, thermal, and durability characteristics. The utilization of industrial wastes in the production of geopolymer materials has both economic and environmental advantages [16]. The geopolymerization process has been investigated as an eco-sustainable technological approach for tailings treatment and heavy metal immobilization [11]. Luevano-Hipolito et al. [17] investigated the methodology for the preparation of the geopolymer materials fabricated with the recycling of waste materials in alternative self-cleaning building materials depending on the fly ash and TiO_2_ content, as well as temperature. The research aimed to reduce building maintenance costs, reduce CO_2_ emissions, and reuse waste. Understanding of the geopolymerization process is of crucial importance for the application of geopolymers for pollutant removal and sustainable construction.

Geopolymers are obtained by the chemical reaction of aluminosilicate minerals with a concentrated alkali solution and the creation of a calcium silicate hydrate (C-S-H) and sodium-aluminosilicate-hydrate (N-A-S-H) gel [18] as a new amorphous to semi-crystalline aluminosilicate phase [19,20].

The raw material dissolves in an alkaline solution, generating Si and Al ions that produce gels. Subsequent reactions of dissolution–coagulation, coagulation–condensation, condensation–polymerization restructuring, and polycondensationresult in the creation of a stiff three-dimensional network composed of SiO_4_ and AlO_4_ tetrahedrons linked by oxygen bridges. The negative charge is balanced by the positive ions (e.g., K^+^, Na^+^, Ca^2+^, and Li^+^) present in the framework of cavities. The basic empirical formula for the geopolymer structure is M_n_[-(SiO_2_)_z_-AlO_2_]_n_·wH_2_O where *M* represents the monovalent alkali metal cation (Ca^2+^, Li^+^, K^+^, or Na^+^), and *n* is the polycondensation degree and indicates the number of SiO_4_ tetrahedra [12].

In accordance with that, SiO_2_- and Al_2_O_3_-containing materials can be used as raw materials for preparing geopolymers. As silicon and aluminum constitute approximately 75% of the Earth’s crust, many rocks and minerals can be used as sources for producing geopolymerization precursors [21]. Using mining tailings as a precursor to produce geopolymers is a promising solution for their comprehensive utilization. Many articles have been published on this topic in the last two decades.

The Si/Al ratio is one of the main factors that determine the formation of gels and the geopolymer properties, including mechanical strength, immobilization capacity, and durability [22,23]. Aseniero et al. [24] also found that the highest compressive strength was achieved when a geopolymer was derived from mine tailings with a Si/Al ratio close to the optimum value of 3.0. After 7 days of curing at ambient temperature, the compressive strength reached about 5.48 MPa. Zhang et al. [25] reduced the extremely high Si/Al ratio of copper tailings by adding various contents of fly ash up to its optimum range (1–3), resulting in a denser microstructure.

The influence of the content of NaOH, as an alkali activator, on the dissolution of Si and Al species, followed by polycondensation, was also observed. The NaOH content also plays a role in determining the Na/Al ratio. The authors recorded the highest compressive strength of 5.32 MPa at a 11% moisture content and 11 M NaOH. The optimum Na/Al ratio for this was found to be 0.79, which is close to the value of about 1.0 reported by Ren et al. [26].

Mining tailings have a low reactivity in alkaline solutions due to their high crystallization degree which results in slow settings, poor durability, and low mechanical strength when used as a single geopolymerization precursor. This limits their application. To improve the properties of the synthesized geopolymers, it is advisable to add aluminosilicate materials with higher reactivity.

Low and non-Ca-containing additives, such as fly ash and zeolite, exhibit higher reactivity than mining tailings during alkali activation. Therefore, more reactive Si and Al can dissolve to form the geopolymer nanostructure, which modifies the resulting strength [18]. The compressive strength for the geopolymer obtained from copper tailings with coal fly ash and the alkaline activator of 10 M NaOH after 28 days of curing at ambient temperature reached about 37 MPa. In this case, most of the tailings can only serve as an aggregate due to their extremely low contribution to reactive Si and Al.

The calcium content in the system significantly affects the properties of the geopolymer at an early age. It results in faster bonding and higher compressive strength of the material compared to systems that do not contain calcium. This is due to the formation of additional hydration gels, such as calcium silicate hydrate (C-S-H) and calcium silicate aluminate hydrate, which are reported to form in the Na_2_O-CaO-SiO_2_-Al_2_O_3_-H_2_O system. These phases can improve the dissolution of aluminosilicates in alkaline solutions and, in turn, enhance the geopolymerization reaction [27,28]. Moreover, the pretreatment (mechanical, thermal, and alkali fusion) of mining tailings is one way to increase their reactivity.

In general, the alkaline interaction of mining tailings is low, and this is the most substantial factor to consider when incorporating tailings into geopolymers. As indicated in research by He et al. [15], there are a number of factors that influence the mechanical properties, compressive strength, and flexural strength of various kinds of tailings used as a geopolymer source. The use of tailings as a precursor for geopolymers depends on the nature of their previous treatment. Pretreatment makes the material available for a faster rate of geopolymerization and results in better mechanical properties of the material. Mechanical, thermal, and thermochemical pretreatments can be used to make mine tailings more reactive, and all can be used for this purpose. Reducing the particle size and expanding their surface area for alkaline dissolution enhances the participation of the mine tailings in the geopolymerization process of mineral components. Moreover, geopolymerization with thermally treated materials (slag or fly ash) has its priorities due to their reactivity, and copper tailings are commonly coupled with other alumina-silicate source materials, such as fly ash, for the production of geopolymers. The copper tailings powder near the interfacial zone actively participates in the geopolymerization reaction with the formation of gel C-(A)-S-H products in an alkaline environment, and also has a filling effect when incorporated as micro powders [29]. The geopolymerization process of copper tailings is continuous and slow at lower curing temperatures (up to 45 °C), while, at higher curing temperatures (≥75 °C), the process of the depolymerization and cracking of the material occurs with a longer curing time. The optimal curing temperature of 60 °C can be used for the production of FT-based geopolymers [30]. The use of copper tailings in the geopolymerization process also achieves the effective immobilization of heavy metals and mitigates their impact on the environment. In addition to the physical mechanism of metal encapsulation, some metals form a covalent bond with the unbridgeable oxygen in the geopolymer, and others can be immobilized within the geopolymer via ionic bonding [31].

This study aims to investigate the influence of the flotation tailings content on geopolymeric material based on natural zeolite, Na-modified zeolite, and fly ash. This is achieved by analyzing the structure and properties of the mentioned geopolymers using instrumental methods such as XRD, SEM-EDS, and TGA/DTA analysis. The mechanical properties of geopolymers were analyzed on hardened samples and as a function of the initial molar ratios of Ca/Si, Si/Al, Na/Al, and H_2_O/Na_2_O. The computer program PHREEQC was used for the modeling of a variety of geochemical reactions for a system including dissolving and precipitating phases to achieve equilibrium with the aqueous phase, and the effects of curing temperature. The pollutant’s leachability was assessed by using the standard method, TCLP.

## 2. Materials and Methods

### 2.1. Materials and Chemicals

The flotation tailings (FTs) used in this study for production of geopolymers are obtained from the Majdanpek copper mine (Serbia). In order to improve the characteristics of geopolymers, fly ash (FA) from thermal power plants in Serbia (TE “Morava”-Svilajnac) and natural zeolite (NZ) from the Zlatokop site (Vranjska Banja, Serbia) were applied as additives.

Sodium chloride (NaCl, p.a.; Zorka Pharma, Šabac, Serbia) was used for the modification of natural zeolite. Natural zeolite was mixed with 2.0 mol·dm^−3^ NaCl solution (× *g* zeolite/100 mL solution) at 65 °C on a magnetic stirrer (RCT basic IKAMAG) with a stirring speed of 650 rpm during 48 h. The obtained sodium-modified natural zeolite was separated from the suspension by vacuum-filtration, washed with distilled water until there was a negative reaction to chloride ions (negative test with AgNO_3_ solution), and left to dry in an oven at 100 °C overnight. Na-modified natural zeolite contains 180 mmol Na per 100 g of zeolite. Sodium hydroxide (NaOH, p.a.; Erba Lachema, Brno, Czech Republic) was used as the alkali activator.

A detailed elemental and mineralogical analysis were performed for all raw materials (flotation tailings, natural zeolite, and fly ash). The results of the characterization are shown in Table 1 and Table 2.

The elemental analysis was conducted by dissolving the material in aqua regia and measuring the concentrations of elements using the AAS technique (Atomic Absorption Spectroscopy).

The results of the chemical composition analysis indicated the highest percentage of Si in all three components (FT, NZ, and FA). Moreover, the flotation tailings contain significant amounts of Fe (10.3 wt.%), S (8.16 wt.%), Ca (10.1 wt.%), Mg (3.01 wt.%), and C (3.05 wt.%). In addition to silicon, zeolite contains the most Al (8.99 wt.%), Ca (3.50 wt.%), and Na (2.52 wt.%). In fly ash, the dominant contents are Si (22.9 wt.%) and Ca (22.2 wt.%), while the content of Al and Fe is 7.14 wt.% and 3.85 wt.%, respectively.

Mineralogical analysis of raw materials confirmed the presence of Si in the form of SiO_2_ and Al in the form of Al_2_O_3_. Iron is present in two phases (FeS and Fe_2_O_3_) depending on the type of raw material. FeS is contained in flotation tailings, while Fe is found in zeolites and fly ash in the form of Fe_2_O_3_. Loss on ignition at 950 °C was 9.1, 10.5, and 11.7% for samples FT, NZ, and FA, respectively.

### 2.2. Sample Preparation

Three series of the geopolymer specimens were prepared by hand-mixing of the flotation tailings with NZ, sodium-modified natural zeolite (NaZ), or FA, separately. Sodium hydroxide solution (10.0 g NaOH/25 cm^3^ H_2_O) was used as the alkali activator. The activating solution is tempered to room temperature before use. Compositions of geopolymer mixtures are given in Table 3.

The fresh paste specimens were cast in molds of 20 mm × 20 mm × 20 mm and vibrated for 5 min to remove entrained air bubbles. The molds with the samples were kept in polyethylene bags to avoid excessive evaporation for 1 day, sealed, and cured in an oven at 60 °C for 1 more day. After this time, they were taken out of the oven, allowed to cool, and aged at 25 ± 2 °C for 28 days.

### 2.3. Samples Analysis

Samples of raw material (NZ, FA, and FT) were analyzed by the XRD and SEM-EDS methods. The determination of particle size distribution for the raw materials based on the SEM image was carried out using the Image Pro Plus 6.0 Software.

Geopolymer samples with the highest share of tailings (50%), aged for 28 days, were analyzed by XRD, SEM-EDS, and TG/DTG methods. The hardened specimens were demolded at 7/14/28 days and tested for compressive strength.

The effects of Ca/Si, Si/Al, and Na/Al molar ratios and geochemical modeling using PHREEQC 3.7.3 software were applied to analyze the mechanical properties of obtained geopolymers. The stability of the elements of the environmental concern within geopolymer structure was investigated by standard leaching tests (TCLP).

### 2.4. Methods

#### 2.4.1. XRD

Mineralogical analyses by X-ray powder diffractometry (XRD) were applied to identify and monitor the main components in geopolymer specimen using powder diffractometer “PHILIPS” (Philips, Amsterdam, The Netherlands), model PW-1710, with the characteristic CuKα radiation (λ = 1.54178 Å) and a scintillation detector, at 40 kV and 30 mA, over the 2Θ range 4–65°, in steps of 0.02° and within the time of 1 s.

#### 2.4.2. SEM-EDS

Scanning electron microscopy (SEM) method was carried out using a JEOL JSM–7001F field emission scanning electron microscope (SEM) (JEOL, Tokyo, Japan) equipped with an INCA energy-dispersive X–ray analysis unit (EDS). An acceleration voltage of 20 kV was used. The analyzed samples were coated with gold (15 nm-thick layer, with density of 19.32 g/cm^3^). For quantitative analyses, the following synthetic standards were used: FeS2 (FeKα, SKα), ZnS (Zn Kα, S Kα), Ni (Ni Kα), Co (Co Kα), Cu (Cu Kα), InSb (Sb Kα), Ag2Te (AgLα), CdS (Cd Lα), HgS (Hg Mα), PbS (Pb Mα), Bi (Bi Mα), CuFeS2 (Cu Kα, Fe Kα), ZnS (Zn Kα), Mn (Mn Kα), Gadolinium Gallium Garnet (Ga Kα), Ge (Ge Lα), InAs (As Kα, In Lα), SnO2 (Sn Lα), PbS (Pb Mα), and Bi (Bi Mα). EDX detection limit is 2σ~0.3 wt.%.

#### 2.4.3. TG/DTG

Thermal analysis was performed using an SDT Q-600 simultaneous TG/DTG-DSC instrument (TA Instruments, New Castle, DE, USA). The samples were heated in an air stream at a flow rate of 100 cm^3^ min^−1^, at a heated rate of 20 °C min^−1^, in the temperature range of 25 to 950 °C.

#### 2.4.4. Unconfined Compressive Strength Measurement

Mechanical properties of geopolymer specimens were assessed by determining its unconfined compressive strength (UCS) after 7, 14, and 28 days of curing. UCS was measured using a hydraulic-testing-machine-type Shimadzu UH-F1000Kni (Shimadzu, Kyoto, Japan) with a clip speed of 2 mm·min^−1^. The results were the mean measurement values of the three samples.

#### 2.4.5. Leaching Methods

A standard leaching method, toxicity characteristic leaching procedure (TCLP), was used to determine leaching of flotation tailing and hardened geopolymer specimens as granular waste under the experimental conditions specified hereafter.

Toxicity characteristic leaching procedure (TCLP) was used to determine whether there are hazardous elements present in waste. The pH of acetic acid was adjusted to 2.88 ± 0.05 before leaching experiments. The crushed samples and acetic acid solution were placed into beakers with a liquid/solid ratio of 20 and extracted for 18 h [32].

After filtration (Millipore, Burlington, MA, USA, 0.45 μm), leachates were acidified to pH 2 with HNO_3_, and atomic absorption spectroscopy (AAS), using Varian SpectrAA 55B device (Varian Inc., Lake Forest, CA, USA ), was used to determine the content of the leach solution.

#### 2.4.6. Geochemical Modeling (PHREEQC Program)

PHREEQC is used for geochemical calculation between equilibrium solids, liquids, and gases. It is a geochemical modeling program capable of simulating kinetically controlled reactions, performing speciation and saturation index calculations, transport calculations, and many other types of reaction calculations using appropriate databases. In this paper, the Cemdata database (http://www.empa.ch/cemdata, accessed on 10 November 2024) was applied. This database includes a comprehensive selection of hydrated phases encountered in geopolymer structure, as well as the calcium (alkali) aluminosilicate hydrate (C-(N-)A-S-H) gel, whoseformation is expected in alkali-activated binders.

In order to perform geochemical modeling, geopolymer samples after 28 days of aging were leached with distilled water, with a L/S ratio of 10, at a temperature of 70 °C. The resulting solutions were filtered immediately after leaching using filter paper (Millipore 0.45 μm). After measuring pH and Eh values, the solutions were acidified to pH < 2 and sent for ICP-OES analysis. Results of the solutions analyses were the input data into PHREEQC and the program calculated the saturation index (SI) for all the possible minerals (in the database) that can be formed within the given components in the solutions.

SI is defined as SI = log (IAP/Ksp), where IAP is the ion activity product, and Ksp is the equilibrium solubility product.

PHREEQC indicates that minerals are in stable form if the saturation index equals 0.0 (zero point zero). Following an initial analysis, if a solid phase has a negative SI, it is assumed to be under-saturated and will dissolve into the solution. Conversely, if the SI is positive, the solution is over-saturated, and a solid phase may form.

## 3. Results and Discussion

### 3.1. Phase Composition of Raw Materials and Obtained Geopolymers (XRD Analysis)

The morphological analysis of the raw material (FT, NZ, and FA) and geopolymer products were characterized by XRD analysis, to investigate the progression of the synthesis reaction and to monitor the type and the quality of the formed products.

**Figure 1:**
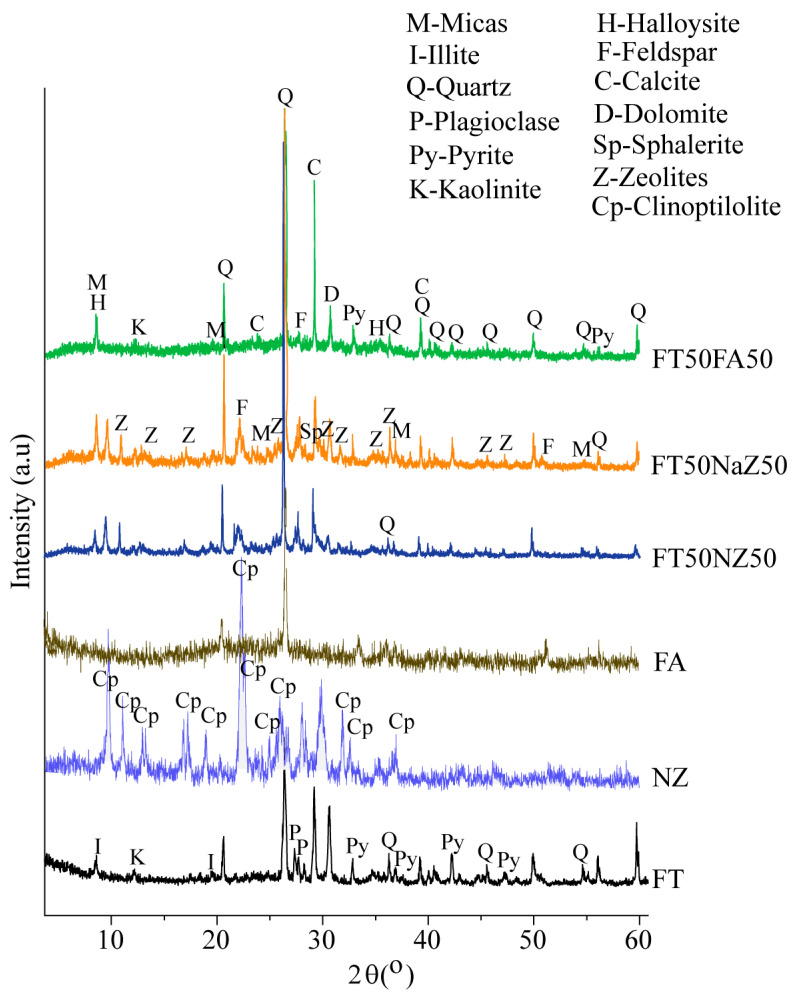
X-ray patterns of the flotation tailings (FT, NZ, and FA) and geopolymers FT50NZ50, FT50NaZ50, and FT50FA50.

The most abundant minerals in the analyzed flotation tailings sample identified by X-ray diffraction analysis (Figure 1) were quartz, pyrite, and carbonates (calcite and dolomite), followed by feldspar, clay minerals, and illite, which were less abundant. The results of the semi-quantitative analysis of the data obtained by XRD were as follows: quartz ≈ 50–65%, total carbonates ≈ 20–25%, kaolinite ≈ 5–10%, pyrite ≈ 5%, and feldspar, clay minerals, and illite (total amount ≈ 20%). The dominant presence of plagioclase was determined from feldspar and kaolinite from clay minerals. The high pyrite content supported the results of the chemical analysis and also indicated the high potential of exposed tailings to generate AMD. The content of carbonate minerals (20–25%) indicated the acid neutralization capacity of the material. The concentrations of Mn (0.14%), Cu (0.072%), Zn (0.086%), and Pb (0.0079%) indicated the possibility of the leaching of these metals from tailings in contact with acidic water [33].

The XRD analysis of NZ is shown in Figure 1. The semi-quantitative analysis of data obtained by XRD showed that it contains 72% clinoptilolites, while feldspar (14%), quartz (12%), clay minerals, and illite (2%) were the main impurities. Figure 1 also shows the diffractogram of FA powder. The X-ray diffraction on a polycrystalline powder sample of FA determined the presence of quartz and plagioclase minerals. The dominant content of quartz minerals confirmed the chemical analysis, which showed that SiO_2_ is the most abundant oxide.

The mineral phase characterization of the geopolymers was performed on samples that had been cured for 28 days. Figure 1 depicts the XRD patterns of alkali-activated NZ-, NaZ-, and FA-based geopolymers with an FT maximal content (50%).

In the geopolymer sample FT50NZ50, the intensity of the quartz peak is the greatest, due to the presence of this mineral in both phases, in the FT as well as in the NZ (Figure 1). According to the results, the most abundant minerals are clinoptilolite and quartz, with less carbonates (calcite is more dominant than dolomite) and mica, while feldspars (plagioclases) and clays are present in traces. The presence of the sulfides (pyrite and sphalerite) is insignificant.

The powder diffraction pattern of the FT50NaZ50 geopolymer sample shows the structural analysis of the geopolymer sample prepared in the presence of Na-modified natural zeolite (Figure 1). No significant changes are observed compared to the sample with unmodified zeolite, which leads to the conclusion that the larger amount of sodium in the zeolite structure does not affect its crystallinity and phase structure.

In the analyzed sample of the geopolymer FT50FA50obtained in the presence of fly ash, the presence of the following minerals was determined: quartz, mica, calcite, dolomite, kaolinite, halloysite, feldspar, and pyrite. The most abundant minerals are quartz, less carbonates, calcite, and then mica, while feldspars (plagioclases) and clays are the least presented. Pyrite is also present in this sample but in traces.

The addition of the NZ, NaZ, and FA does not affect the crystallinity of the FT (Figure 1). Based on the presented X-ray diffraction patterns, a significant difference cannot be observed between the newly formed geopolymer materials.

### 3.2. SEM-EDS Analysis of Raw Materials and Obtained Geopolymers

The size and morphology of the samples of the raw materials and the obtained geopolymers were observed by the scanning electron microscopy method (SEM), while EDS analysis was used to qualitatively and semi-quantitatively determine the elemental composition.

**Figure 2:**
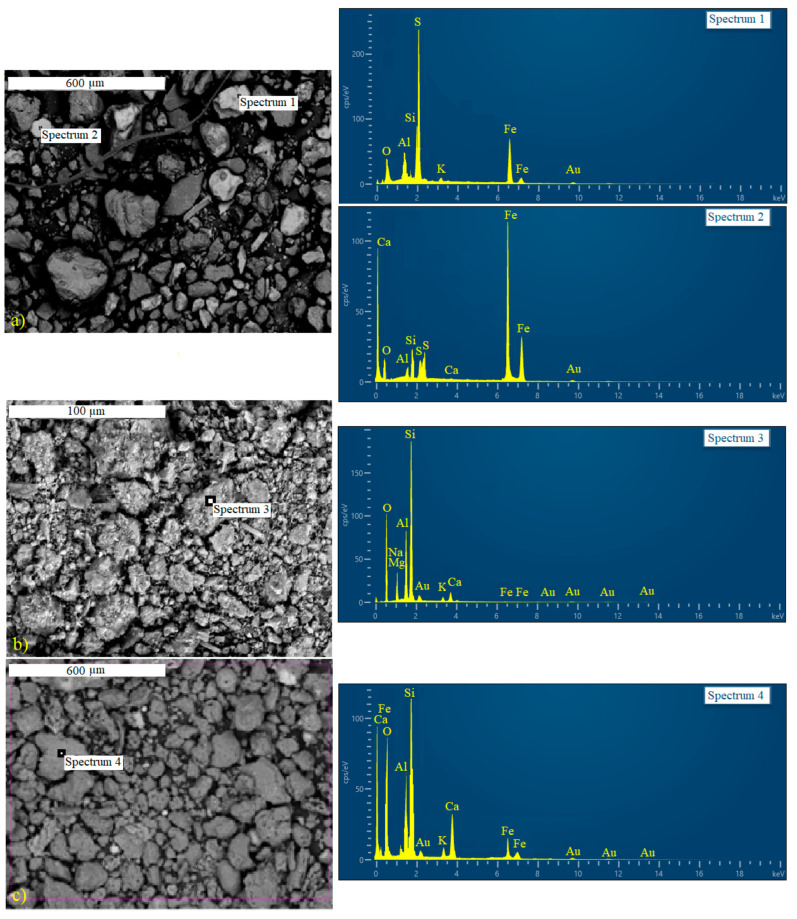
SEM-EDS analysis of the raw samples: (**a**) FT, (**b**) NZ, and (**c**) FA.

Micrographs of raw materials (FT, NZ, and FA) obtained by scanning electron microscopy with the spectra of elements represented in the samples are shown in Figure 2. Moreover, the particle size distribution for the samples was carried outin SEM images of the samples using the Image Pro Plus Software.

Particles of different sizes, shapes, and elemental composition can be observed on the micrographs of the FT sample (Figure 2a). This indicates a heterogeneous content that, in addition to aluminosilicates, also includes sulfide ores. Particle sizes ranged from 9.3 μm to 162.12 μm. The analysis indicated a mean particle size of 47.37 μm with a narrow distribution between 30–60 μm. A few larger particles (e.g., 153.6 μm and 162.12 μm) were also observed. The micrograph of zeolite powder (Figure 2b) shows irregular-shaped different-sized particles and their aggregated structure. The zeolite particle sizes ranged from 1.58 μm to 25.70 μm. The analysis showed a mean particle size of 8.05 μm with a narrow distribution between 5–6 μm. Particles in a larger range of up to 25.70 μm, which may represent aggregates of the sample, were also identified. The micrograph of the FA sample (Figure 2c) reveals that the material consists of particles of irregular shape, heterogeneous structure, and different size. The fly ash particle sizes ranged from 8.85 μm to 129.81 μm. The analysis indicated a mean particle size of 47.13 μm with a narrow distribution between 12–60 μm. A few larger particles above 72 μm (e.g., 126.64 μm) were observed. In EDS analyses of the samples, different characteristic peaks are observed in the spectrum, indicating the dominant presence of Si, Al, Ca, and Fe, thereby confirming the results of the chemical analysis of raw materials.

**Figure 3:**
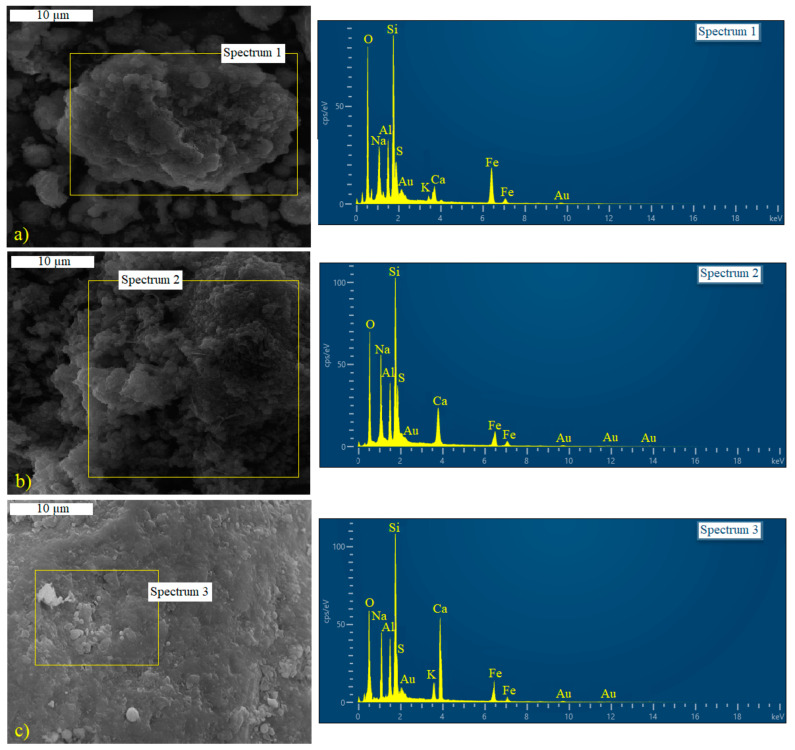
SEM-EDS analysis of the geopolymers: (**a**) FT50NZ50, (**b**) FT50NaZ50, and (**c**) FT50FA50.

Characteristic SEM-EDS images of the obtained geopolymers (FT50NZ50, FT50NaZ50, and FT50FA50) are shown in Figure 3. The micrograph of the FT50NZ50 sample, presented in Figure 3a, shows a massive hydration product in the center surrounded by a porous structure, caused by the incomplete hydration. The micrograph of the FT50NaZ50 sample (Figure 3b) also shows a porous structure, similar to the FT50NZ50 sample, however, with a denser structure and with a more developed gelatinous hydration product. In contrast to zeolite-based geopolymers, the micrograph of FT50FA50 geopolymers (Figure 3c) shows a well-developed gel-like hydration product of the geopolymer matrix with a high degree of homogeneity. From themicrographs and results of EDS analyses, it can be seen that the hydrated structure and gel-like phase corresponds to the C-(N-)A-S-H gel. Moreover, the structure of the geopolymer matrices becomes more compact and denser with increasing Ca content, that is, the Ca/Si ratio, which, in zeolite-based geopolymers, is 0.2, while, in geopolymers with fly ash, it is 0.4. The increase in the density of the hydrated matrix is also influenced by the Na/Al ratio, which is higher in the FT50NaZ50 (1.5) sample with a more compact structure than in the FT50NZ50 (Na/Al 1.2) sample. However, in the FT50FA50 geopolymer sample with the most developed hydrated structure, this ratio is the lowest (Na/Al 1.1). This indicates that the molar ratios of gel-forming elements (Si, Al, Ca, and Na) have to be optimized.

### 3.3. TG-DTG Analysis of Geopolymers

The thermogravimetric analysis of flotation tailings (FTs) and geopolymer products (FT50NaZ50 and FT50FA50) was investigated by heating all samples in the atmosphere of air at a temperature range of 20–950 °C.

**Figure 4:**
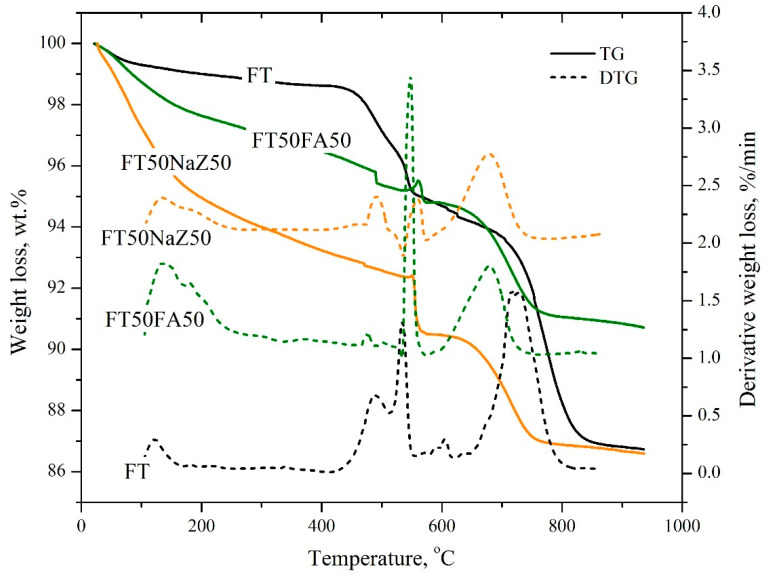
TG and DTG curvesof samples flotation tailings (FTs) and geopolymer products FT50NaZ50 and FT50FA50.

Figure 4 shows that samples of flotation tailings and geopolymer products with Na-modified zeolite had a similar mass loss, 13.3 to 13.4 wt.%, respectively. However, the total mass loss for geopolymer products with fly ash (FT50FA50) was lower (9.3 wt.%).The reason for this is that the geopolymer FT50FA50 contains components that are products of thermal processing (fly ash) which have been unchanged.

**Figure 5:**
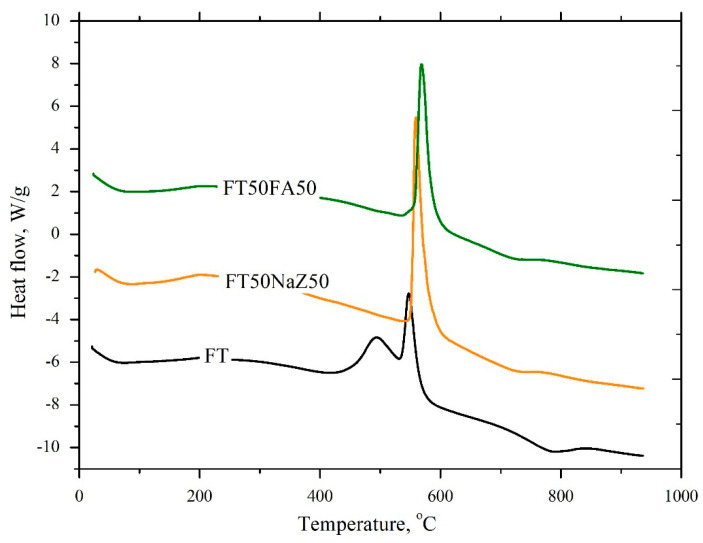
DSC curves for samples of flotation tailings (FTs) and geopolymer products FT50NaZ50 and FT50FA50.

By comparing the TG, DTG, and DSC curves of all three tested materials (raw FT and geopolymers with a 50% FA and 50% NaZ; Figure 4 and Figure 5), it is evident that the thermal decomposition occurs in several stages. The first stage presents the loss of free and chemically bound or adsorbed water [34]. In the case of row FTs, the surface water completely evaporates at up to 100 °C with a mass loss of about 1 wt.% and the DTG maxima is at 46 °C (Figure 4). In the case of geopolymers, this stage lasts until up to 200 °C and the DTG maxima is at 64 °C for both materials, with a mass loss of 6.5 and 2.5 wt.% for FT50NaZ50 and FT50FA50, respectively. This can be attributed to the dehydration of hydrated phases such as ettringite and calcium silicate hydrate (C-S-H) [34,35]. The greater mass loss in the FT50NaZ50 geopolymer in this temperature interval may indicate a greater amount of hydrated phases; however, it may also be associated with a greater amount of adsorbed water within the zeolite structure.

The next step in the thermal decomposition of all three samples is in the temperature interval from 425 to 600 °C. In the case of the row FT sample, it represents the decomposition of carbon compounds in the flotation tailings, and the presence of several peaks indicates the existence of several different carbon compounds [36]. The rate of mass loss of 1.5 wt.% is detected at 487 °C, and a mass loss of 2.0 wt.% at 542 °C (Figure 4). The thermal decomposition of raw tailings inside the geopolymers is reflected in their DTG curves. In the case of the FT50FA50 sample, the mass loss of the geopolymer samples can be attributed to the dehydroxylation of the mineral matter, as well as to the collapse and disintegration of the zeolite framework structure [35]. The DTG maxima of the geopolymers is shifted to higher temperatures (570 and 560 °C for FT50FA50 and FT50NaZ50, respectively) in comparison with the sample of row FTs (the DTG maxima is at 540 °C). From the TG curves, it can be seen that, at a temperature of about 570 °C, there is a small increase in the mass of the geopolymer prepared in the presence of the FA, which could be attributed to the oxidation reactions in the air stream and the formation of a dense geopolymer matrix. This indicates that the geopolymers are more thermally stable than the FT itself.At higher temperatures (ranging from 600 up to 950 °C), the mass loss of row FTs as well as the geopolymers can be attributed to the carbonation of the mineral phases present in the flotation tailings [36].

The amount of the exchanged heat during the decomposition of the row FTs as well as of the geopolymer is shown in Figure 5. Exothermic peaks in the temperature interval from 425 to 600 °C were observed on all three curves, which indicate that heat is released during tailings decomposition. The decomposition of row FTs occurs in two steps with the release of 420 J/g of heat (the maxima are at 490 and 548 °C). In the case of the geopolymer samples FT50NaZ50 and FT50FA50, the DSC maxima were slightly shifted to higher temperatures (560 and 569 °C, respectively). The released amount of heat was 577 J/g for FT50NaZ50 and 465 J/g for the FT50FA50 sample. Compared to the FT and Na-modified zeolite-based geopolymer materials, FA-based materials present advantages with a slight shift of DSC peaks to higher temperatures and a slower heat of hydration. Moreover, endothermic peaks (at around 65 and 770 °C) were observed at all three DSC curves and can be attributed to the dehydration and decarbonization.

### 3.4. Mechanical Properties of Geopolymer

**Figure 6:**
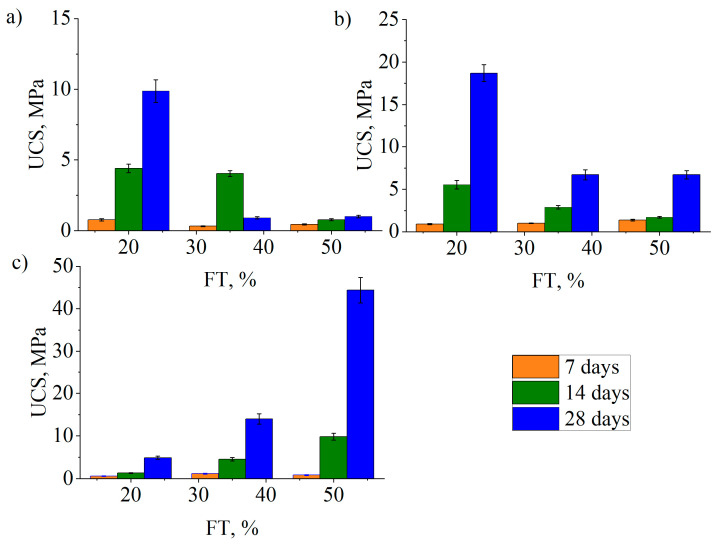
Unconfined compressive strength of geopolymer based on (**a**) NZ, (**b**) NaZ, and (**c**) FA with FT content 20 wt.%, 35wt.%, and 50 wt.%, aged 7, 14, and 28 days.

The effectiveness of the flotation tailings’ geopolymerization with zeolite (natural and modified) and fly ash was evaluated by examining the mechanical properties of the obtained geopolymers. The results of the compressive strength (UCS) tests of geopolymers aged 7, 14, and 28 days are shown in Figure 6a–c. The results of the Ca/Si, Si/Al, and Na/Al molar ratios are shown in Table 4. Their effects on the mechanical properties of zeolite- and fly-ash-based geopolymers with flotation tailings were analyzed.

Figure 6 shows the compressive strength of NZ- (Figure 6a), NaZ- (Figure 6b), and FA-based geopolymers (Figure 6c) with different FT contents at different curing times and the same curing temperature (70 °C, 24 h).

The results (Figure 6a–c) showed a significant increase in the compressive strength of geopolymers with time (7, 14, and 28 days), with the highest measured values for geopolymers aged 28 days. The most notable difference in the measured compressive strength values was observed for geopolymers aged 28 days with a higher proportion of FTs. That is in accordance with the positive effects of the curing time and temperature on the mechanical properties of geopolymer materials. The curing temperature ranging from 25 to 80 °C promotes a more uniform distribution of C-S-H and C-(N)-A-S-H gels and enhances the strength of tailings-based geopolymers [37]. Accordingly, in this study, the geopolymer curing temperature was 70 °C for the first 24 h.

With the addition of FTs from 20 wt.% to 50 wt.%, the UCS of zeolite-based geopolymers (Figure 6a,b) decreased, reaching the maximum when 20 wt.% FTs was added. With the increase in tailings content in this system, the molar ratios of Si/Al and Ca/Si also increase, from 2.7 to 3.1 and from 0.1 to 2, respectively. With the increase in these molar ratios, an increase in UCS was expected; however, this was not the case. This indicates that FTs in the geopolymer system with zeolite and sodium hydroxide as an alkaline activator remain inert.

The compressive strength of Na-modified-zeolite-based geopolymers (Figure 6b) with 20 wt.% FT contents curing at 7, 14, and 28 days were 0.9 MPa, 5.5 MPa, and 18.7 MPa, respectively, which exceeded the compressive strength of natural-zeolite-based geopolymers (Figure 6a) under the same curing conditions. This is a consequence of the higher Na/Al molar ratio (up to 1.4) in the geopolymer system with Na-modified zeolite and tailings, which positively affects the solubility of the system and the density of the geopolymer structure [15].

With the addition of FT increasing from 20 wt.% to 50 wt.%, the compressive strength of fly-ash-based geopolymers (Figure 6c) increased and reached the maximum when 50 wt.% FT was added. The compressive strength of fly-ash-based geopolymers with 50 wt.% FT contents achieved at 7, 14, and 28 days were 0.9 MPa, 9.8 MPa, and 44.4 MPa, respectively, which exceeded the compressive strength of zeolite-based geopolymers (Figure 6a,b) under the same curing conditions. These compressive strength measurement results indicate a better activation of the flotation tailings in the fly ash geopolymer system. The obtained result is correlated with an increase in the Si/Al molar ratio from 2.7 to 3.1; and a potential increasein Si-O-Si bonds and residual silica was possible. Moreover, the molar ratios of Ca/Si were higher than in systems with zeolite. However, as the proportion of tailings increased, the Ca/Si molar ratio decreased. Despite this decrease, the unconfined compressive strength (UCS) of the material with fly ash increased. This can be attributed to the potential coexistence of C-(N-)A-S-H phases [38], which contributes to the formation of a multiphase composite structure within the fly-ash-based geopolymers. This structure enhances the material’s stability and results in a denser geopolymer matrix. This shows that the compressive strength of the fly-ash-based geopolymer could be improved by replacing the FA with appropriate amount of the FT. Similar results were obtained by testing the compressive strength of a geopolymer obtained from copper tailings with coal fly ash and an alkaline activator of 10 M NaOH after 28 days of curing at an ambient temperature that reached about 37 MPa [37].

The Si/Al, Na/Al, and Ca/Si ratios are the main factors that determine the geopolymer properties (the mechanical strength, immobilization capacity, and durability) [22,23]. The analyzed systems had an Si/Al molar ratio ranging from 2.7 to 3.1, as shown in Table 4. The geopolymer sample FT50FA50 had an Sl/Al ratio of 3.1 and the highest measured compressive strength value. All these values are close to the values of 3.0 and 3.31 performed by Aseniero et al. [24] and Manjarrez and Zhang [39]. The optimum Na/Al ratio for the investigated geopolymers was found to be 1.1, which is close to the value of about 1.0 reported by Ren et al. [26] and Zhang et al. [40]. The Ca/Al ratio had the greatest influence on the compressive strength of the geopolymer. The analysis of the results showed that the optimal value of this molar ratio is 0.4 for the fly-ash-based geopolymer with the highest proportion of FTs (50 wt.%). The calcium content in the system significantly affects the properties of the geopolymer at an early age. This results in the faster bonding and higher compressive strength of the material compared to systems that do not contain calcium. This is due to the formation of additional hydration gels, such as calcium silicate hydrate (C-S-H) and calcium silicate aluminate hydrate, which are reported to form in the Na_2_O-CaO-SiO_2_-Al_2_O_3_-H_2_O system. These phases can improve the dissolution of aluminosilicates in alkaline solutions and, in turn, enhance the geopolymerization reaction [27,28].

### 3.5. Geochemical Modeling in PHREEQC Program

The chemical composition of the solutions after leaching the geopolymer samples with distilled water are shown in Table 5. The concentrations of the elements of interest (Al, Ca, Fe, K, Mg, Na, S, and Si) together with the pH and Eh values were the input data for the geochemical modeling in the PHREEQC program.

**Figure 7:**
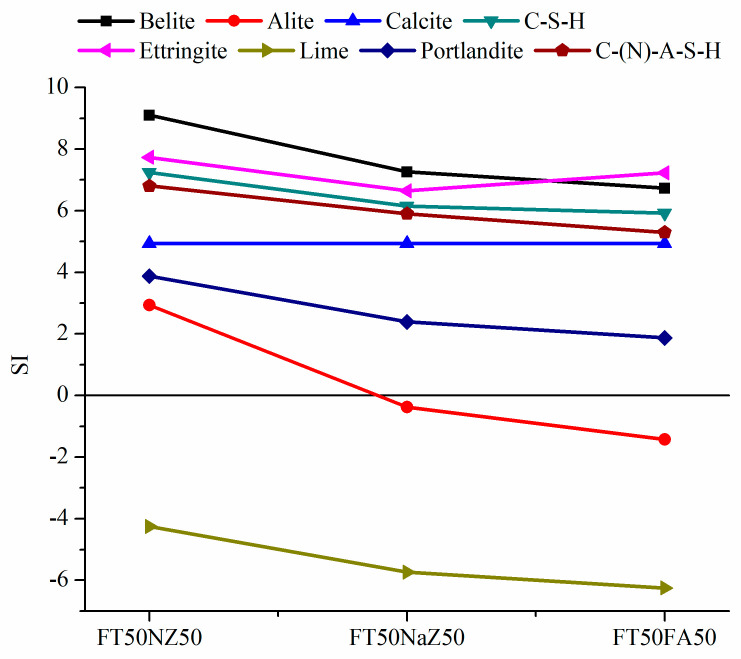
SI values of hydrated and mineral phases potentially formed in the geopolymer systems.

The output data from the geochemical modeling were the SI values of the phases that are potentially formed in the geopolymer system. The results are graphically presented in Figure 7 as the possible formation or dissolution of hydrated and mineral phases in the geopolymer samples expressed through the SI values. The presence of hydrated phases, primarily C-S-H and C-(N)-A-S-H gels, is responsible for the development of compressive strength in geopolymers. From Figure 7, it can be seen that the value of the SI for the C-S-H gel is slightly higher for geopolymers with fly ash (FT50FA50), 7.24, compared to geopolymers with zeolite, 6.15 for FT50NaZ50 and 5.92 for the FT50NZ50 sample. Interestingly, a similar case can be found for the C-(N)-A-S-H phase formed by the alkali activation of the binder, 6.8 for FT50FA50 compared to 5.9 and 5.3 for FT50NaZ50 and FT50NZ50, respectively. This means that alkali activation using an NaOH solution has a greater effect on fly ash compared to zeolite as additives in the geopolymerization process. The dissolution of lime (CaO), shown by negative values for the SI, is followed by the formation of portlandite (Ca(OH)_2_) as a product of the hydration process, and, again, is more pronounced in the FT50FA50 sample. The possibility of ettringite formation, expressed through the values of the SI, varies in the three observed systems, 7.73, 6.65, and 7.23 for FT50FA50, FT50NaZ50, and FT50NZ50, respectively, and it is mainly limited by the availability of sulfate ions [41]. An important fact is that the SI value for calcite (CaCO_3_) is the same for all three samples and it is 4.94. This means that the calcite mineral phase from the composition of the flotation tailings remained inert in the geopolymerization process. This confirms the role of FTs as a filler in geopolymer systems [42,43]. The presence of the mineral phases alite and belite are one of the main factors in the process of hydration and the development of compressive strength [44,45]. The presence of belite is possible in all three systems, again with a slightly greater possibility in the geopolymer with fly ash (SI 9.10) than with zeolite (7.26 in FT50NaZ50 and 6.73 in FT50NZ50). The most obvious difference in the observed geopolymer samples is the positive value of the SI for the mineral alite in FT50FA50 and its negative value for geopolymers with zeolite. The main reason for this is that these calcium–silicate minerals are originally found in the composition of fly ash and are responsible for the development of the hydrated structure and higher compressive strength in the FT50FA50 geopolymer [44].

### 3.6. Leaching Behavior of Elements from Raw Materials and Obtained Geopolymers

The presence of various heavy metals in flotation tailings is a potential environmental problem due to their leaching into the soil and groundwater. Geopolymerization is considered one of the sustainable methods for the treatment of tailings containing toxic elements, and leaching behavior is an important indicator of the efficiency of the immobilization of heavy metals in geopolymers.

**Figure 8:**
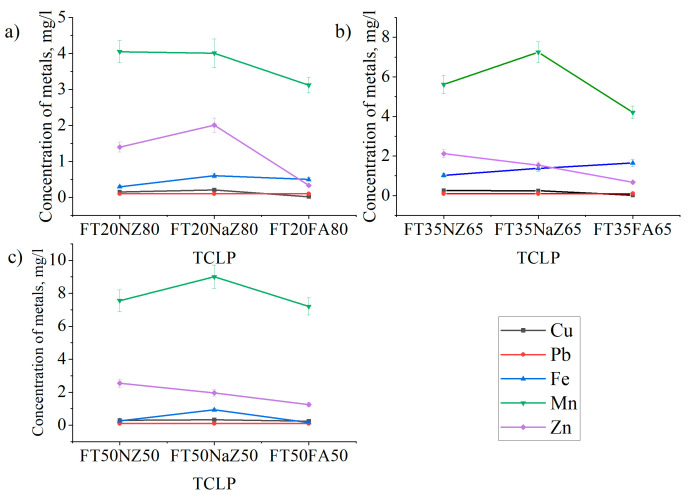
Concentrations of heavy metals in leachate after TCLP test on (**a**) natural zeolite, (**b**) Na-modified zeolite, and (**c**) fly-ash-based geopolymer.

In this study, the leaching behavior of elements (Cu, Pb, Fe, Mn, and Zn) from raw materials (FT, NZ, NaZ, and FA) and the zeolite- and fly-ash-based geopolymer was analyzed using the standard method of TCLP. The concentrations of contaminants and Eh–pH values measured in the leachates for the toxicity characteristics (TCLP) are shown in Table 6 and Figure 8.

The heavy metal contents in the leaching solution from the TCLP test of the geopolymer samples were lower than those of the raw materials used in the synthesis. It is evident that all the measured values for the elements (Cu, Pb, Fe, Mn, and Zn) are significantly lower than the limit values for the TCLP test (Table 6). The ability to successfully immobilize heavy metals contained in flotation tailings within the geopolymer based on zeolites and fly ash was demonstrated by these results. Although an increase in the tailings content in the prepared samples led to an increase in the concentration of the elements in the leaching solution, these values remained within acceptable limits and the tested geopolymers have the characteristics of non-toxic and stable non-reactive materials.

The results presented in Table 6 indicated the more intensive leaching of Mn from tailings and raw materials (NZ, NaZ, and FA) compared to other elements. In addition to the leaching of Mn from tailings (29.02 mg/L), the leaching of Mn from fly ash (30.89 mg/L) was dominant. A significant decrease in the concentration of this element in TCLP leachate from FA-based geopolymers (Figure 8) indicated its effective immobilization. The mechanism of immobilization of heavy metals in the geopolymer is their good encapsulation in the three-dimensional geopolymer network. It has been confirmed that the encapsulation of Mn in the three-dimensional geopolymer network is accompanied by the formation of stable compounds of the new phase MnSiO_3_, which leads to a decrease in the leaching concentration of Mn [46]. The release of Mn in the leaching test indicates the potential microporosity of the material and leaching from the degraded geopolymer matrix.

Finally, it was observed that the leachability of metals (Cu, Pb, Zn, Fe, and Mn) was significantly reduced due to immobilization in geopolymers. The metal immobilization process is the most effective in geopolymers based on fly ash with the largest share of flotation tailings.

## 4. Conclusions

A geopolymer based on zeolite and fly ash with different contents of flotation tailings was prepared by mixing with an alkaline solution (sodium hydroxide). Effects of the flotation tailings content on the structure, mechanical properties, and chemical stability of the obtained geopolymers were investigated. The main conclusions could be drawn as follows:

- The optimal mixture proportion was 50% flotation tailings and 50% fly ash, with initial molar ratios of Si/Al 3.0, Na/Al 1.1, and Ca/Si 0.4, and the highest measured compressive strength of 44.3 MPa after 28 days of curing.
- Increasing the tailings content by up to 50% in zeolite-based geopolymers had a negative effect on the compressive strength of the material due to the inertness of the tailings and the low calcium content in the system (Ca/Si ≤ 0.3),despite the optimal molar ratios of Si/Al 3.1 and Na/Al 1.1.
- The increase in the tailings content by up to 50% in geopolymers based on fly ash had a positive effect on the compressive strength of the material. The higher concentration of Ca in the system (Ca/Si ≥ 0.3) promotes the formation of a multiphase composite structure inside the fly-ash-based geopolymers, improving its structural stability, and forming a dense geopolymer matrix. The optimal values of Si/Al and Na/Al in this system were 3.1 and 1.1, respectively.
- Geochemical modeling using PHREEQC has demonstrated that fly ash is a more effective additive for the geopolymerization of flotation tailings compared to zeolite. The presence of reactive calcium–silicate minerals in fly ash contribute to a better hydration structure development and higher unconfined compressive strength (UCS).
- All tested geopolymers have the characteristics of non-toxic and stable non-reactive materials.
- The metal immobilization process is the most effective in geopolymers based on fly ash with the largest share of flotation tailings.
- This study demonstrated that FTs, as mining waste from the separation and beneficiation processes of ore, can be effectively reused in fly-ash-based geopolymers, producing a sustainable material with a high compressive strength and non-hazardous characteristics, adding value to this waste.

Future research about the utilization of FT in geopolymer materials will include the following:

- The impact of FT particle geometry on the microstructure, hydration, workability, strength, and durability of the obtained materials;
- The impact of different activation agents (sodium/potassium silicate and potassium hydroxide) and curing temperature on the characteristics of the obtained geopolymers;
- The evaluation of the long-term durability of the material using standard tests such as the chloride permeability, water permeability, and resistance to various environmental factors (alkali–silica reaction, corrosion, carbonation, acid/salt attack, creep, and fire resistance). This assessment will help predict the practical applications of the resulting geopolymer materials.

## Figures and Tables

**Table 1:** The chemical composition of FT, NZ, and FA.

Element	FT, wt.%	Element	NZ, wt.%
Si	29.12	Si	32.1
Fe	10.34	Al	8.99
Ca	10.11	Ca	3.50
S	8.16	Na	2.52
C	3.05	Fe	1.75
Mg	3.01	K	1.37
Al	0.69	Mg	0.42
Mn	0.14		
**Element**	**FT, mg** **·kg^−3^**	**Element**	**FA, wt.%**
Cu	662.12	Si	22.91
Zn	921.43	Ca	22.24
Pb	93.20	Al	7.14
Sb	55.34	Fe	3.85
Cr	25.22	Mg	0.57
Ni	25.04	S	0.21
As	25.15	Mn	0.12
Co	21.31		
Ba	20.01		

**Table 2:** Mineralogical composition of FT, NZ, and FA.

Phase	FT, wt.%	NZ, wt.%	FA, wt.% DS *
FeS	4.21	/	/
Fe_2_O_3_	3.58	2.40	8.14
Na_2_O	1.26	3.25	0.29
SiO_2_	45.61	65.42	37.03
CaO	6.21	4.70	23.41
CaCO_3_	20.70	/	/
Al_2_O_3_	11.14	16.31	10.14
K_2_O	2.69	3.16	0.86
MgO	3.35	0.68	0.72
SO_3_	/	/	0.97
H_2_O	1.19	3.16	13.44

* DS—dry substance content.

**Table 3:** Compositions of geopolymer mixtures.

Geopolymer	FT		NZ		NaZ		FA	
	wt.%	g	wt.%	g	wt.%	g	wt.%	g
FT20NZ80	20	10	80	40.0	-	-	-	-
FT20NaZ80	20	10	-	-	80	40.0	-	-
FT20FA80	20	10	-	-	-	-	80	40.0
FT35NZ65	35	17.5	65	32.5	-	-	-	-
FT35NaZ65	35	17.5	-	-	65	32.5	-	-
FT35FA65	35	17.5	-	-	-	-	65	32.5
FT50NZ50	50	25	50	25.0	-	-	-	-
FT50NaZ50	50	25	-	-	50	25.0		
FT50FA50	50	25	-	-	-	-	50	25.0

**Table 4:** The initial molar ratio of Ca/Si, Si/Al, and Na/Al.

Geopolymer	Initial Molar Ratio		
	Si/Al	Na/Al	Ca/Si
FT20NZ80	2.7	1.1	0.1
FT35NZ65	2.9	1.1	0.1
FT50NZ50	3.1	1.2	0.2
FT20NaZ80	2.7	1.4	0.1
FT35NaZ65	2.9	1.4	0.1
FT50NaZ50	3.1	1.4	0.2
FT20FA80	2.4	1.0	0.6
FT35FA65	2.7	1.0	0.5
FT50FA50	3.0	1.1	0.4

**Table 5:** Concentration of elements in solutions after leaching the geopolymers samples, in mg/L.

Element	FT50FA50	FT50NaZ50	FT50FA50
Al	19.54	19.54	17.61
Ca	140.81	140.80	335.41
Fe	0.51	0.51	0.732
K	142.70	142.72	/
Mg	4.41	4.40	5.97
Na	193.92	237.02	167.75
S	19.15	19.47	19.01
Si	6.37	6.37	5.41
pH	11.44	11.53	12.22
Eh, mV	−232.91	−235.95	−274.72

**Table 6:** Concentrations of heavy metals in TCLP leachates of raw materials with pH–Eh values of leachates for zeolite- and fly-ash-based geopolymer.

Parameter	FT	NZ	NaZ	FA	Ref. Values
Cu, mg/L	4.61	≤0.1	≤0.1	0.03	25
Pb, mg/L	≤0.1	≤0.1	≤0.1	≤0.1	5
Fe, mg/L	2.25	0.21	0.25	0.35	-
Mn, mg/L	29.02	1.79	1.99	30.89	-
Zn, mg/L	9.15	0.53	0.71	1.64	250
pH	5.12	3.96	3.54	5.39	
Eh, mV	111.4	178.21	156.21	96.62	
		**FT20NZ80**	**FT20NaZ80**	**FT20FA80**	
pH		5.51	5.27	6.91	
Eh, mV		92.71	108.32	99.91	
		**FT35NZ65**	**FT35NaZ65**	**FT35FA65**	
pH		5.44	5.23	6.34	
Eh. mV		87.21	108.34	40.12	
		**FT50NZ50**	**FT50NaZ50**	**FT50FA50**	
pH		5.77	5.64	6.73	
Eh. mV		79.44	87.04	79.41	

## Data Availability

The original contributions presented in this study are included in the article. The data further supporting this study's findings are available from the author, Marija Štulović, upon reasonable request.
